# Reconsidering Digoxin in Atrial Fibrillation: From Historical Controversy to Physiologically Guided and Personalized Rate Control

**DOI:** 10.3390/biomedicines13123098

**Published:** 2025-12-16

**Authors:** Silvia Ana Luca, Adelina Andreea Faur-Grigori, Cristina Văcărescu, Dragos Cozma, Simina Crișan, Dan Gaiță, Mihai-Andrei Lazăr, Constantin-Tudor Luca

**Affiliations:** 1 Doctoral School, “Victor Babeș” University of Medicine and Pharmacy, 300041 Timișoara, Romania; silvia.luca@umft.ro (S.A.L.); andreea.faur@umft.ro (A.A.F.-G.); 2 Institute of Cardiovascular Diseases Timisoara, 300310 Timisoara, Romania; dragos.cozma@umft.ro (D.C.); simina.crisan@umft.ro (S.C.); dan.gaita@umft.ro (D.G.); lazar.mihai@umft.ro (M.-A.L.);; 3 Research Center of the Institute of Cardiovascular Diseases Timisoara, 300310 Timisoara, Romania; 4 Department of Cardiology, “Victor Babeș” University of Medicine and Pharmacy, 300041 Timisoara, Romania

**Keywords:** atrial fibrillation, digoxin, heart failure, rate control, serum digoxin concentration, confounding by indication

## Abstract

**Background**: The role of digoxin in atrial fibrillation, particularly in patients with heart failure, has long been debated. Observational studies reporting higher mortality have fueled skepticism, yet growing evidence suggests that these findings largely reflect prescription bias, confounding by indication, and inadequate adjustment for serum-level rather than intrinsic toxicity. **Objective**: To reassess digoxin’s role in atrial fibrillation with heart failure using contemporary evidence and to propose a physiology-based, personalized monitoring framework. **Evidence review**: We reevaluated the studies that initially linked digoxin to excess mortality and reassessed these associations through three analytic pillars: randomized evidence, bias deconstruction, and exposure–response relationships. Across datasets, low serum digoxin concentrations were consistently associated with stable resting rate control without increasing mortality. **Key findings**: Low-dose, continuously administered digoxin is a viable second-line option for atrial fibrillation rate control in patients who are hypotensive or intolerant of β-blockers. Safety is concentration-dependent; adverse outcomes increase at higher serum digoxin concentration (≥1.2 ng/mL). Resting heart rate can serve as a contextual surrogate of exposure: persistent HR > 100 bpm in stable patients usually reflects underexposure rather than digoxin toxicity, whereas bradycardia should prompt immediate serum digoxin concentration testing. **Proposal**: A probability-based monitoring model that integrates heart rate, renal function, dosage, electrolytes, and drug–drug interactions to guide when serum digoxin concentration measurement is warranted. As a future direction, a supervised “pill-in-the-pocket” supplemental dose strategy could be evaluated for transient tachycardia in selected, stable patients. **Conclusions**: When properly dosed and contextually monitored, digoxin remains a safe, effective, and individualized rate-control option in atrial fibrillation with heart failure. Prospective validation of probability-guided monitoring and evaluation of a “pill-in-the-pocket” approach could simplify digoxin management while maintaining safety.

## 1. Introduction

Atrial fibrillation (AF) is among the most prevalent cardiac arrhythmias worldwide and a major driver of cardiovascular morbidity and mortality [1]. Contemporary management focuses on rhythm control, ventricular rate control, and anticoagulation to reduce stroke and heart-failure related complications. While β-blockers and non-dihydropyridine calcium-channel blockers remain first-line agents for both acute and chronic rate control, digoxin is still a therapeutic option, particularly in older adults, in patients with hypotension, and in those with coexisting heart failure with reduced ejection fraction (HFrEF) [2,3]. Current clinical evidence on digoxin in AF pertains to continuous low-dose therapy, supported by monitoring for efficacy and safety [4,5].

For more than two centuries, since Withering’s 1785 description, digoxin has endured alternating cycles of enthusiasm and skepticism [6]. Several large observational studies, most notably TREAT-AF, reported an association between digoxin use and increased mortality in AF, raising concerns about its safety profile [7]. However, subsequent analyses suggest that these findings are largely explained by prescription bias and residual confounding, as digoxin has often been prescribed to older, clinically vulnerable patients with advanced heart failure [8].

More recent randomized and population-based data have challenged the notion of inherent harm. RATE-AF, the first head-to-head randomized comparison of low-dose digoxin versus bisoprolol, showed comparable rate control, fewer adverse effects, and lower NT-proBNP with digoxin over 12 months, supporting the editorial view that appropriately titrated, monitored low-dose digoxin is a reasonable second-line option when first-line agents are not tolerated or are insufficient [9,10]. Complementing these findings, a nationwide nested case–control analysis suggested that the “mortality signal” is unlikely to be causal, noting parallel associations with non-cardiac outcomes (sepsis, institutionalization) consistent with digoxin as a marker of clinical vulnerability rather than a mediator of harm [11].

Despite a steady decline in prescribing, reflecting persistent safety concerns rather than new evidence of harm [8,12,13], emerging data now support a more focused, evidence-based role for low-dose, continuously administered digoxin in hemodynamically fragile patients and in those intolerant of β-blockers [14].

This review traces digoxin’s journey from historical controversy and observational bias to contemporary evidence supporting physiologically guided, personalized rate control in AF. We synthesize randomized, propensity-adjusted, and meta-research data to clarify the true meaning of the so-called “mortality signal”, and we introduce a forward-looking model that uses heart rate (HR) as a contextual surrogate for serum digoxin concentration (SDC) to guide pragmatic monitoring and, in selected patients, a potential “pill-in-the-pocket” strategy. Furthermore, we ask the contextual question central to clinical practice and guidelines: does current evidence justify upgrading digoxin to a higher class of indication, particularly in patients with AF and HFrEF who are hypotensive, β-blocker-intolerant, or inadequately rate-controlled?

## 2. Pharmacology of Digoxin

Digoxin is a cardiac glycoside whose principal action is the selective and reversible inhibition of the membrane Na^+^/K^+^-ATPase in cardiomyocytes. By raising intracellular Na^+^, this blockade reduces the driving force for the Na^+^/Ca^2+^ exchanger, allowing Ca^2+^ to accumulate within the cell and the sarcoplasmic reticulum. With each subsequent depolarization, more Ca^2+^ is released to bind troponin C, thereby augmenting myocardial contractile force (positive inotropy) [15]. Beyond its myocyte effects, digoxin potentiates vagal input at the SA and AV nodes. This results in negative chronotropy and negative dromotropy by slowing AV-nodal conduction and increasing refractoriness. Together, these actions support its usefulness for ventricular rate control in AF, particularly at rest [16].

Clinically, digoxin has a narrow therapeutic window, requiring careful exposure management. Exposure must be managed carefully [17]. Most AF and HF studies did not measure serum digoxin concentrations, which limits causal inference regarding safety. A meta-analysis of 19 major studies reported serum levels in only three trials (one AF, two HF), showing an exposure-outcome evidence gap [18] (AF: [19]; HF: [20,21]). Across datasets with measured levels, the concentration–risk relationship was consistent. Post hoc analyses and clinical studies converge on a conservative target window of ~0.5–0.9 ng/mL, with outcomes worsening above ~1.0 ng/mL [22]. The balance of pharmacologic and clinical findings favors lower therapeutic serum levels. In anticoagulated AF cohorts, higher exposure was associated with greater platelet activation and higher urinary thromboxane B_2_ levels. Concentrations < 1.0 ng/mL conferred no excess risk compared with patients not receiving digoxin, whereas >1.2 ng/mL was linked to elevated cardiovascular risk [23].

After oral administration, approximately 70–80% of digoxin is absorbed in the proximal small intestine. Its binding to serum albumin is modest (~20–30%), and tissue distribution is extensive, consistent with a large apparent volume of distribution. After ingestion, digoxin reaches peak serum concentrations at ~1–3 h, then undergoes a tissue distribution phase that equilibrates by ~6–8 h (the recommended window for serum concentrations sampling), followed by predominantly renal elimination with a half-life of 36–48 h, prolonged in chronic kidney disease, so that the steady state is typically achieved after 5–7 days of continuous dosing [24].

Uptake into the myocardium determines the pharmacodynamic effects, and the severity of toxicity correlates with both serum and tissue concentrations. Elimination depends largely on renal function; among patients with end-stage renal disease on hemodialysis, higher all-cause mortality has been observed during digoxin therapy, with the lowest risk at serum concentrations < 0.9 ng/mL, whereas small dose increases can precipitate severe toxicity (reported levels ~4.9 ng/mL) [25,26].

In practice, selective concentration monitoring is justified: obtain serum digoxin concentrations ≥ 6–8 h after a dose (ideally at trough) and recheck after dose changes, shifts in renal function, or the introduction/withdrawal of P-gp modulators [27]. Recommended targets aim to maintain low, continuous levels within a conservative range (HFSA suggests < 1.0 ng/mL, preferably 0.7–0.9 ng/mL), and major AF guidelines support targeted monitoring in higher-risk scenarios, including initiation, dose adjustments, renal impairment, polypharmacy, or suspected toxicity.

## 3. Current Perspectives on Chronic Digoxin Therapy in Atrial Fibrillation with Heart Failure

### 3.1. Current Guideline Positioning and Evolving Class of Recommendation

In contemporary practice, the role of digoxin in patients with AF and HF remains selectively valuable, but variably positioned across guidelines. The 2024 European Society of Cardiology (ESC) guidelines assign digoxin a Class IIa recommendation for long-term rate control in AF patients with HF, particularly in those with reduced left ventricular ejection fraction or limited tolerance to β-blockers or nondihydropyridine calcium-channel blockers. The guideline emphasizes its effectiveness in controlling resting heart rate, particularly in sedentary patients, while acknowledging its reduced efficacy during exertion [28].

Similarly, the 2023 ACC/AHA/ACCP/HRS atrial fibrillation guideline recommends digoxin as a Class IIa indication for ventricular rate control in AF, particularly for patients with HFrEF, hypotension, or intolerance to first-line agents such as β-blockers or calcium-channel blockers [29]. In the heart-failure domain, the 2022 AHA/ACC/HFSA guideline lists digoxin as a Class IIb recommendation in symptomatic HFrEF despite optimal guideline-directed medical therapy (GDMT), with the aim of reducing HF hospitalizations [30].

These classifications reflect a cautious but supportive stance toward digoxin use in carefully selected patients. Digoxin remains largely considered a second-line or add-on therapy. However, contemporary evidence and clinical experience show that it remains useful in patients intolerant to first-line agents, hemodynamically fragile, or still symptomatic despite standard therapy. These insights highlight the need to better define the clinical scenarios in which digoxin provides the greatest benefit.

### 3.2. Reassessing Digoxin Through Contemporary Evidence: A Clarified Evidence-Based Role

The contemporary case for chronic digoxin therapy in AF with HF is supported by a growing body of evidence demonstrating that its historical controversy largely stems from methodological bias rather than intrinsic drug toxicity. The rationale for reconsidering digoxin’s therapeutic position rests on three major pillars: (1) randomized trials, (2) clarification that reported mortality “signals” result from confounding, and (3) heart-failure data showing morbidity reduction at safe serum digoxin concentrations.

Pillar 1: Randomized Evidence in Atrial Fibrillation: The RATE-AF trial provided the first modern head-to-head randomized comparison of low-dose digoxin versus bisoprolol in older adults with permanent AF and HF symptoms. Over 12 months, digoxin achieved similar resting rate control, fewer adverse effects, and lower NT-proBNP levels compared with bisoprolol [9]. Although RATE-AF remains the only large RCT directly comparing the two agents, earlier crossover studies by Lewis [31], Matsuda [32], and Farshi [33] confirmed that digoxin effectively controls the resting ventricular rate but is less effective during exertion due to its lack of sympatholytic activity. Importantly, combination therapy with β-blockers or calcium-channel blockers produced optimal rate control without increased adverse events, supporting digoxin as an evidence-based second-line therapy when first-line agents are contraindicated or not tolerated.

Pillar 2: Understanding the “Mortality Signal”: Associations between digoxin and increased mortality have been repeatedly observed in large observational studies, such as the TREAT-AF registry and the post hoc analysis of digoxin use within the AFFIRM trial [34,35]. However, these cohorts disproportionately included older, frailer patients with advanced HF and hypotension, particularly those in whom digoxin is preferentially prescribed when β-blockers or calcium-channel blockers are unsuitable. This prescribing pattern introduces confounding by indication and prescription bias. In contrast, the contemporary ORBIT-AF registry analysis by Allen et al. found that digoxin use was associated with higher mortality only among patients without heart failure. In contrast, outcomes in those with HF were neutral. These findings further underscore the susceptibility of observational datasets to residual confounding and treatment-selection bias [36].

Supporting this interpretation, a propensity-matched analysis of the AFFIRM trial by Gheorghiade et al. showed no excess mortality or hospitalization in patients receiving digoxin once the baseline characteristics were balanced [37]. Further emphasizing the limitations of non-randomized evidence, the meta-analysis by Ouyang et al. synthesized data from more than 300,000 AF patients and demonstrated a modest association between digoxin use and higher mortality. However, because all included studies were observational and lacked serum level measurements, the authors acknowledged that residual confounding remained the most plausible explanation for the observed mortality signal [38].

The umbrella review by Gazzaniga synthesized 12 prior meta-analyses. They revealed major heterogeneity, a predominance of observational data, and a critically low study quality [39]. Furthermore, Baker et al. emphasized that most studies failed to adjust for SDC, an important determinant of safety, which likely leads to an overestimation of the reported risk [40]. Collectively, these meta-research findings showed that the mortality signal likely reflects residual confounding, rather than intrinsic drug toxicity [41].

Table 1 summarizes representative studies illustrating how these biasing mechanisms influence the reported relationship between digoxin and mortality.

Notably, Singh et al. demonstrated that when rigorous propensity matching and comprehensive covariate adjustment are applied, the association between digoxin use and mortality disappears [42]. This finding exemplifies that once bias is minimized, digoxin’s effect becomes neutral or beneficial, confirming that the observed mortality excess in prior studies is primarily a reflection of bias within study design, rather than a toxic effect.

**Table 1:** Summary of key studies illustrating bias-driven mortality associations with digoxin in AF.

Study (Year)	Study (Type)	Population	Selection Bias	Information Bias	Mortality Outcome	Bias Impact on Results
**Whitbeck (2013)** [43]	Post hoc observational analysis within the AFFIRM RCT	4060 AF patients (2153 on digoxin)	Digoxin not randomized; sicker patients more likely to receive digoxin	Multivariate adjustment; no SDC data;	↑ all-cause, CV, arrhythmic mortality	Residual confounding likely due to preferential use of digoxin in higher-risk patients
**Washam (2015)** [44]	Post hoc observational analysis within ROCKET-AF RCT	14,171 AF patients (5239 on digoxin)	Digoxin not randomized; digoxin group had more HF and comorbidities	RCT dataset with independent, blinded adjudication of outcomes; no SDC data	↑ all-cause, ↑ CV mortality; ↑ SCD in HF patients	Associations may reflect prescription bias and confounding by indication (digoxin users had worse HF, renal dysfunction, and comorbidities)
**Eisen (2017)** [45]	Post hoc observational analysis within ENGAGE AF-TIMI 48 RCT	21,105 AF patients (6327 on digoxin)	Digoxin not randomized; more often in patients with advanced HF and multiple comorbidities	residual confounding despite multivariable adjustment; no data on SDC;	↑ all-cause mortality; ↑ CV mortality;↑ SCD;	Higher mortality probably reflects confounding by indication;
**Lopes (2018)** [46]	Post hoc observational analysis within the ARISTOTLE RCT	17,897 AF patients (5824 on digoxin)	Digoxin use not randomized; users had higher comorbidity burden and worse clinical status	SDC available but single measurement; possible residual confounding	SDC ≥ 1.2→ ↑ all-cause mortality (56% higher); each +0.5↑in SDC →+19% mortality; new digoxin initiation→ ↑ SCD	Mortality risk appears strongly related to higher serum digoxin levels and may be amplified by confounding by indication; a causal effect cannot be excluded but is not definitively proven;
**Elayi (2020)** [47]	Post hoc, observational secondary analysis within AF-CHF RCT	1376 patients with AF + HFrEF	Digoxin not randomized; more often used in patients with more severe HF and lower EF	No SDC data; possible residual confounding despite advanced modeling	↑ all-cause mortality, ↑ cardiac mortality, ↑ arrhythmic death; no significant effect on cardiac hospitalizations	Mortality differences likely reflect confounding by HF severity and lack of serum level data rather than a proven causal drug effect
**Singh (2020)** [42]	Propensity-matched observational registry (OPTIMIZE-HF + Medicare)	1768 matched patients with HF + AF	Non-randomized; new digoxin users balanced for 56 variables via propensity matching	Registry-based data; no SDC levels; residual confounding possible	No ↑ all-cause mortality; ↓ HF readmissions; results consistent in HFrEF and HFpEF	Propensity matching reduces measurable bias; any residual confounding is unlikely to overturn the neutral mortality findings

Abbreviations: AF, atrial fibrillation; HF, heart failure; HFrEF, heart failure with reduced ejection fraction; CV, cardiovascular; SCD, sudden cardiac death; SDC, serum digoxin concentration; RCT, randomized controlled trial; symbols: ↑, increased; ↓, decreased.

Pillar 3: Heart-Failure Evidence and the Importance of Exposure: The Digitalis Investigation Group (DIG) trial remains the pivotal randomized study evaluating digoxin in chronic HF (sinus rhythm, LVEF ≤ 45%). Over a median of 37 months, digoxin had no effect on all-cause mortality (34.8% vs. 35.1%, *p* = 0.80) but significantly reduced HF hospitalizations and deaths from pump failure [20]. Subsequent analyses revealed a strong dose–response relationship: SDCs of 0.5–0.9 ng/mL were associated with lower mortality and hospitalization, whereas levels ≥ 1.2 ng/mL markedly increased the risk of mortality, particularly among elderly and female patients [48].

This exposure–safety pattern was confirmed in the ARISTOTLE trial (n = 17,000), where SDC ≥ 1.2 ng/mL conferred a 56% higher mortality risk, and each 0.5 ng/mL increase in SDC raised mortality by 19%, regardless of HF status [46]. These findings establish that the safety of digoxin is concentration-dependent, and that therapeutic benefit is preserved when serum levels are maintained within the lower range (0.5–0.9 ng/mL).

### 3.3. Interpretation and Clinical Implications

When analyzed through the lens of modern evidence, the case against digoxin weakens substantially. Excess mortality reported in observational studies is primarily explained by selection bias, confounding by indication, and inadequate serum levels adjustment rather than intrinsic toxicity. At low serum concentrations and with appropriate monitoring, digoxin provides reliable resting rate control, reduces HF morbidity, and remains particularly valuable in patients with HFrEF, low blood pressure, or intolerance to β-blockers.

Taken together, randomized, propensity-adjusted analyses, and meta-analytic data support a clearer, context-specific role for digoxin in guideline-directed therapy. However, given the absence of new large-scale randomized trials and the recent publication of major AF and HF guidelines, the available evidence does not currently justify upgrading its class of recommendation. Current evidence highlights the need to refine the clinical contexts in which digoxin offers the greatest benefit.

## 4. Proposal: Heart Rate as a Contextual Surrogate Marker for Serum Digoxin Concentration in Chronic Therapy

In contemporary digoxin management, SDC remains the cornerstone for dose titration and safety monitoring. However, SDC measurement is often limited by delayed laboratory turnaround, cost, and availability, particularly in ambulatory or resource-limited settings. We propose a physiologically grounded, context-based approach in which resting HR serves as a dynamic surrogate marker to estimate the probability of high or low SDC.

### 4.1. Physiologic Premise

Digoxin’s principal pharmacodynamic effect in AF is vagotonic slowing of AV nodal conduction [49,50]. Accordingly, the resting ventricular rate reflects, at least in part, the degree of digoxin exposure [51].

Nevertheless, therapeutic, or even elevated SDCs may fail to control ventricular rate when sympathetic activation or systemic illness overrides its vagotonic influence. Conditions such as acute infection or sepsis, decompensated heart failure, thyrotoxicosis, hypoxemia, anemia, and electrolyte disturbances (hypokalemia, hypomagnesemia) can transiently increase sympathetic tone or reduce AV-nodal responsiveness, leading to rapid ventricular rates despite therapeutic SDCs [52,53].

When secondary causes are excluded and renal function and autonomic tone remain stable, the following physiological relationship between HR and SDC can be reasonably inferred:

High resting HR (>100 bpm)—indicates a low probability of supratherapeutic SDC, suggesting underexposure, reduced drug absorption, or elevated sympathetic drive [54];

Controlled HR (60–90 bpm)—likely reflects a therapeutic SDC (0.5–0.9 ng/mL) with effective rate control [55];

Low HR (<60 bpm) or new conduction delay—likely high probability of excessive SDC or emerging toxicity, warranting immediate measurement and clinical reassessment [56].

### 4.2. Probability Framework

Building on these physiological principles, the relationship between SDC and HR can be conceptually interpreted through a probability-based lens. In patients with atrial fibrillation, high SDC typically manifests as bradycardia or varying degrees of AV block. Rapid ventricular rates most often indicate low or subtherapeutic exposure. Thus, the probability that a patient with AF and a resting heart rate above 100 bpm has a supratherapeutic SDC is very low (Table 2).

Elevated digoxin levels are correlated with slower heart rates and an increased risk of conduction delay or bradyarrhythmia, while tachycardia in digoxin-treated patients generally reflects diminished pharmacodynamic effect, poor absorption, or sympathetic predominance [57]. In chronic AF therapy, the probability of elevated SDC when HR > 100 bpm is estimated to be very low (likely < 10%), based on pharmacodynamic trends driven from DIG, ARISTOTLE, and RATE-AF cohorts [4,20,46]. In post hoc analyses of the Digitalis Investigation Group (DIG) trial, SDC values ≥ 1.2 ng/mL were associated with lower ventricular rates and higher all-cause mortality, confirming that bradycardia and conduction delay, not tachycardia, are the typical clinical manifestations of supratherapeutic exposure [20].

These probabilistic associations provide a practical framework for clinical decision-making. In patients with AF chronically treated with digoxin, resting heart rate can therefore function as a contextual marker of digoxin exposure and autonomic balance. Rather than serving as a substitute for serum digoxin measurement, HR may help clinicians stratify the pre-test probability of toxicity or underexposure, guiding when laboratory assessment is truly warranted (Table 2). When renal function, autonomic tone, and drug interactions remain stable, an HR above 100 bpm suggests low digoxin exposure and minimal risk of supratherapeutic concentration, whereas a rate below 60 bpm or new AV conduction delay should prompt immediate biochemical assessment and possible dose reduction [58].

An additional consideration when interpreting digoxin exposure, especially when SDC are unavailable, is the role of ECG markers in the digitalis effect. Classical electrophysiological signatures such as downsloping “scooped” ST-segments, T-wave alterations, QT-interval shortening, and an increased burden of premature ventricular complexes reflect the degree of Na^+^/K^+^-ATPase inhibition and have long been recognized as markers of digitalis effect and early toxicity [59,60]. Integrating these ECG markers into follow-up evaluations could strengthen exposure assessment in chronic digoxin therapy, particularly when probability-based or ‘pill-in-the-pocket’ strategies are considered, and biochemical confirmation is not readily accessible. Contemporary guidelines also emphasize rhythm and conduction monitoring in patients receiving AV-nodal agents, underscoring the potential clinical value of ECG-based assessment when SDC is unavailable [28,30]. Prospective studies should evaluate whether combining ECG morphology with heart-rate trends and Bayesian exposure models enhances the accuracy of predicting therapeutic versus supratherapeutic SDC.

This probability-based logic allows clinicians to rationally prioritize the timing and necessity of SDC determination, optimizing monitoring and reducing unnecessary testing. It is important to emphasize that this HR-guided framework has clear limitations. HR cannot reliably reflect digoxin exposure in the presence of sympathetic activation, intercurrent illness, medication changes, or autonomic variability, even when these appear clinically stable. Moreover, the probability estimates are derived indirectly from exposure-response patterns in previous studies and should be viewed as hypothesis-generating rather than validated clinical thresholds. Prospective studies are required to confirm their accuracy and safety.

### 4.3. Evidence Status and Future Validation

To date, no prospective study has validated HR as a quantitative surrogate for SDC. The current framework is therefore hypothesis-generating and should be tested using Bayesian or logistic predictive models integrating HR, renal function, digoxin dose, potassium, and drug interactions.

From both clinical and research perspectives, this paradigm supports a probability-driven monitoring strategy that merges physiologic and pharmacokinetic inputs into a decision-support algorithm. Prospective validation could employ Bayesian or logistic models. In practical terms, a future Bayesian or logistic model would need to incorporate at minimum resting HR, renal function (eGFR), digoxin dose, potassium level, and major P-glycoprotein drug interactions, with optional inclusion of age, sex, and HF severity. The model’s output would estimate the probability of supratherapeutic (SDC ≥ 1.2 ng/mL) or subtherapeutic exposure, conditional on these clinical variables. Validation would require prospective cohorts of AF patients on chronic digoxin therapy with systematically measured SDC, allowing model calibration and discrimination to be tested against observed serum levels and clinical outcomes.

**Figure 1:**
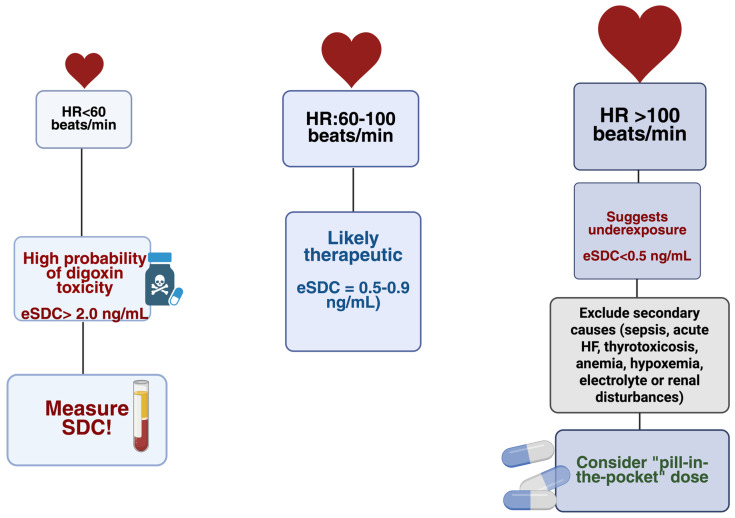
HR-guided probability zones for estimated eSDC: HR < 60 bpm—high likelihood of toxicity; HR 60–100 bpm—likely therapeutic levels; HR > 100 bpm—likely underexposure after excluding secondary causes. eSDC = estimated serum digoxin concentration; HR = heart rate. Created in BioRender. Faur, A. (2025) https://BioRender.com/awxsa7l, accessed on 13 December 2025.

Such modeling could individualize safety thresholds, minimize unnecessary blood testing in stable patients, and ensure timely intervention in those at risk of toxicity [41,61] (Figure 1).

By reframing HR as a contextual physiologic marker rather than a substitute measurement, digoxin monitoring becomes more closely aligned with its pharmacodynamic principles. This approach shifts clinical management from rigid serum-based thresholds toward an adaptive, physiology-driven framework that achieves both safety and practicality in AF.

## 5. Future Perspectives: Toward a “Pill-in-the-Pocket” Strategy for Digoxin

The probability-guided framework naturally suggests the potential for a future “pill-in-the-pocket” approach in selected patients receiving chronic digoxin therapy. In patients chronically treated with digoxin who develop transient resting tachycardia (>100 bpm) despite stable renal function and no precipitating illness (e.g., acute infections, decompensated HF, thyrotoxicosis, electrolyte disturbances, renal dysfunction, anemia or hypoxia), a high HR most often reflects subtherapeutic exposure rather than toxicity. In this setting, a small, carefully titrated supplemental dose administered under clinical supervision could restore rate control without the immediate need for SDC testing. This approach parallels the established “pill-in-the-pocket” rhythm-control strategy, combining pharmacologic precision with individualized adaptability.

Figure 1 summarizes the HR-guided probability zones that estimate whether digoxin exposure is likely subtherapeutic, therapeutic, or excessive. HR < 60 bpm represents a high-risk zone, reflecting the known association between bradycardia, AV-nodal delay, and elevated digoxin levels. HR 60–100 bpm typically corresponds to t therapeutic exposure in stable patients, whereas HR > 100 bpm is most consistent with underexposure once secondary sympathetic triggers are excluded. The figure serves as a conceptual aid rather than a replacement for formal SDC monitoring.

Implementing this strategy in the future would require strict patient selection, integration of clinical context (HR trends, recent doses, renal function), rigorous validation through Bayesian modeling and pragmatic clinical trials. If successful, this approach could reduce unnecessary testing, simplify outpatient digoxin management, and support real-time, physiology-based dose adjustment. Ultimately, a supervised “pill-in-the-pocket” model may represent a step toward more personalized and adaptive rate control in patients with AF and HF.

## 6. Discussion

This review reassesses the long-standing controversy around digoxin in AF by integrating randomized evidence, bias-aware reinterpretation of observational findings, and exposure-response data. Together, these perspectives clarify that appropriately dosed and contextually monitored, digoxin remains a safe and effective option for ventricular rate control in patients with AF and HF.

A central conclusion from contemporary evidence is that the historical “mortality signal” attributed to digoxin is largely artifactual. Large observational cohorts, particularly TREAT-AF, ROCKET-AF, and ENGAGE AF, have reported higher mortality among digoxin users, but these associations were met in populations disproportionately composed of older, frailer patients with advanced HF, renal dysfunction, and hypotension, in whom digoxin was preferentially selected when β-blockers or calcium-channel blockers were unsuitable [7,44,45]. This pattern reflects substantial confounding by indication and prescription bias. Importantly, the signal is not uniform across phenotypes: the ORBIT-AF registry demonstrated that digoxin initiation was associated with substantially increased mortality in patients without heart failure, whereas outcomes in those with HF were neutral. This pattern suggests that the observed risk reflects underlying patient characteristics rather than a consistent drug effect [36].

Furthermore, bias-sensitive analyses, including the propensity-matched AFFIRM evaluation, have shown that once baseline imbalances are corrected, digoxin is not associated with excess mortality or hospitalization [37]. Umbrella reviews and analytic overviews similarly emphasize that residual confounding, unmeasured frailty, and the absence of serum-level adjustment explain much of the historical mortality signal rather than true drug toxicity [39,40].

Digoxin safety is strongly concentration dependent. When maintained within conservative serum ranges (0.5–0.9 ng/mL), digoxin does not increase all-cause or cardiovascular mortality. In contrast, outcomes worsen consistently at SDC ≥ 1.2 ng/mL, underscoring the concentration-dependent nature of digoxin’s safety profile [20,48].

In clinical practice, resting HR may serve as a contextual physiologic marker: persistent HR > 100 bpm in stable patients generally suggests underexposure rather than toxicity, whereas new-onset bradycardia (<60 bpm) or AV-conduction delay warrants immediate SDC measurement and biochemical reassessment [54,55,56].

These principles align with current AF and HF guideline recommendations, where ESC 2024 and ACC/AHA/HRS 2023 classify digoxin as a rate-control option for patients with AF and HF, while the AHA/ACC/HFSA 2022 guideline provides a Class IIb recommendation for reducing HF hospitalizations in symptomatic HFrEF [28,29,30].

Beyond traditional monitoring, digital health technologies may refine this framework. Deep-learning algorithms applied to standard ECGs can detect asymptomatic ventricular dysfunction and evolving AF substrates with high accuracy, revealing latent electrophysiologic signatures not apparent to human readers [62]. Machine-learning models integrating ECG, wearable photoplethysmography, intracardiac electrograms, and clinical variables further improve AF detection, stroke-risk prediction, cardioversion outcomes, and ablation planning [63]. Building on this, AI-enhanced models could potentially estimate digoxin exposure by integrating HR dynamics, ECG morphology, and wearable-derived rhythm data, identifying early signs of subtherapeutic or supratherapeutic effect and guiding safe, timely intervention. Such tools would complement, not replace, SDC monitoring and may eventually support an AI-guided “pill-in-the-pocket” strategy for transient rate acceleration in carefully selected patients [64].

Future studies should clarify the long-term safety and clinical applicability of low-dose, physiologically guided digoxin therapy in AF and HF, and determine how individualized monitoring strategies, including probability-guided assessment and AI-enabled tools, can be integrated into everyday practice. Prospective validation of supervised supplemental dosing (“pill-in-the-pocket”) and exposure-prediction models will be essential to maintain safety while simplifying care. Viewed through contemporary, bias-aware evidence and exposure-response data, digoxin remains a safe, effective, and highly individualizable rate-control option in AF with HF, provided the dose is conservative, the clinical context is respected, and monitoring is optimally applied.

## 7. Conclusions

Digoxin remains a useful and safe option for resting rate control in chronic AF, particularly in patients with hypotension, HFrEF, or intolerance to β-blockers. The historical mortality signal associated with digoxin appears to result largely from confounding, frailty selection, and the absence of serum-level adjustment, rather than from a direct toxic effect. When maintained within conservative therapeutic serum levels, digoxin does not increase all-cause or cardiovascular mortality.

## Figures and Tables

**Table 2:** Relationship between steady-state SDC and resting ventricular rate in patients with AF.

Interpretation	Expected SDC	Typical Ventricular Rate
Optimal therapy	0.5–1.0 ng/mL	70–100 bpm
Subtherapeutic	<0.5 ng/mL	>100 bpm
Toxic	>2.0 ng/mL	<60 bpm

## Data Availability

No new data were created or analyzed in this study.

## Abbreviations

The following abbreviations are used in this manuscript:

ACC	American College of Cardiology
ACC/AHA/ACCP/HRS	American College of Cardiology/American Heart Association/American College of Chest Physicians/Heart Rhythm Society
AHA	American Heart Association
AHA/ACC/HFSA	American Heart Association/American College of Cardiology/Heart Failure Society of America
AF	Atrial Fibrillation
AFFIRM	Atrial Fibrillation Follow-up Investigation of Rhythm Management
ARISTOTLE	Apixaban for Reduction in Stroke and Other Thromboembolic Events in Atrial Fibrillation
AV	Atrioventricular
bpm	Beats per Minute
CKD	Chronic Kidney Disease
DDI	Drug–Drug Interaction(s)
DIG	Digitalis Investigation Group
DHP	Dihydropyridine (non-DHP = non-dihydropyridine)
ECG	Electrocardiogram
EACTS	European Association for Cardio-Thoracic Surgery
eGFR	Estimated Glomerular Filtration Rate
EF	Ejection Fraction
ENGAGE AF–TIMI 48	Effective Anticoagulation with Factor Xa Next Generation in Atrial Fibrillation–Thrombolysis In Myocardial Infarction 48
ESC	European Society of Cardiology
ESRD	End-Stage Renal Disease
GDMT	Guideline-Directed Medical Therapy
HF	Heart Failure
HFpEF	Heart Failure with Preserved Ejection Fraction
HFrEF	Heart Failure with Reduced Ejection Fraction
HFSA	Heart Failure Society of America
HR	Heart Rate
LVEF	Left Ventricular Ejection Fraction
NCX	Sodium–Calcium Exchanger (Na^+^/Ca^2+^ Exchanger)
NKA	Na^+^/K^+^-ATPase
NT-proBNP	N-terminal pro–B-type Natriuretic Peptide
P-gp	P-glycoprotein (ABCB1)
P-gp modulators	Drugs that inhibit or induce P-gp and alter digoxin exposure
PITP	Pill-in-the-Pocket
RCT	Randomized Controlled Trial
ROCKET AF	Rivaroxaban Once Daily Oral Direct Factor Xa Inhibition Compared with Vitamin K Antagonism for Prevention of Stroke and Embolism Trial in Atrial Fibrillation
SA node	Sinoatrial Node
SCD	Sudden Cardiac Death
SDC	Serum Digoxin Concentration
SR	Sarcoplasmic Reticulum
TDM	Therapeutic Drug Monitoring
TREAT-AF	The Retrospective Evaluation and Assessment of Therapies in Atrial Fibrillation
